# “Voluntary in quotation marks”: a conceptual model of psychological pressure in mental healthcare based on a grounded theory analysis of interviews with service users

**DOI:** 10.1186/s12888-022-03810-9

**Published:** 2022-03-17

**Authors:** Sarah Potthoff, Jakov Gather, Christin Hempeler, Astrid Gieselmann, Matthé Scholten

**Affiliations:** 1 Institute for Medical Ethics and History of Medicine, Ruhr University Bochum, Markstraße 258a, 44799 Bochum, Germany; 2 Department of Psychiatry, Psychotherapy and Preventive Medicine, LWL University Hospital, Ruhr University Bochum, Alexandrinenstraße 1-3, 44791 Bochum, Germany; 3 Department of Psychiatry, Charité_Universitätsmedizin Berlin, Campus Benjamin Franklin, Berlin, Germany

**Keywords:** Informal coercion, Treatment pressure, Informed consent, Voluntariness, Perceived coercion, Communication, Qualitative research, Psychiatry

## Abstract

**Background:**

Psychological pressure refers to communicative strategies used by professionals and informal caregivers to influence the decision-making of service users and improve their adherence to recommended treatment or social rules. This phenomenon is also commonly referred to as informal coercion or treatment pressure. Empirical studies indicated that psychological pressure is common in mental healthcare services. No generally accepted definition of psychological pressure is available to date. A first conceptual analysis of psychological pressure focused on staff communication to promote treatment adherence and distinguished between persuasion, interpersonal leverage, inducements and threats.

**Aim:**

The aim of this study was to develop a conceptual model of psychological pressure based on the perspectives of service users.

**Methods:**

Data were collected by means of semi-structured interviews. The sample consisted of 14 mental health service users with a self-reported psychiatric diagnosis and prior experience with coercion in mental healthcare. We used theoretical sampling and contacted participants via mental healthcare services and self-help groups to ensure a variety of attitudes toward the mental healthcare system in the sample. The study was conducted in Germany from October 2019 to January 2020. Data were analyzed according to grounded theory methodology.

**Results:**

The study indicated that psychological pressure is used not only to improve service users’ adherence to recommended treatment but also to improve their adherence to social rules; that it is exerted not only by mental health professionals but also by relatives and friends; and that the extent to which service users perceive communication as involving psychological pressure depends strongly on contextual factors. Relevant contextual factors were the way of communicating, the quality of the personal relationship, the institutional setting, the material surroundings and the level of convergence between the parties’ understanding of mental disorder.

**Conclusions:**

The results of the study highlight the importance of staff communication training and organizational changes for reducing the use of psychological pressure in mental healthcare services.

## Background

The use of coercion in mental healthcare services is controversial and has sparked off public debates around the world and academic debates across disciplines. A common distinction in the literature is that between ‘formal’ and ‘informal’ coercion [1, 2]. Formal coercion refers to interventions carried out against the will of mental health service users, including involuntary hospital admission, involuntary treatment and coercive measures, such as seclusion, mechanical restraint and chemical restraint [3–5]. Informal coercion, on the other hand, refers to communicative strategies used to influence the decision-making of service users and improve their adherence to recommended treatment or social rules. While formal coercion is legally regulated in most countries [6], informal coercion is typically not regulated by law [1, 2]. Moreover, while the various forms of formal coercion are defined clearly, a generally accepted definition of informal coercion is still lacking [1, 2].

Szmukler and Appelbaum [7] developed a first conceptual analysis of what they call “treatment pressures,” distinguishing between persuasion, interpersonal leverage, inducements and threats. Persuasion involves influencing service users’ decision-making by means of rational argumentation; interpersonal leverage involves conditional changes in emotional attitudes within interpersonal relationships; inducements involve conditional proposals to make people better off if they accept the proposal; and threats involve conditional proposals to make people worse off if they refuse the proposal.

**Fig. 1:**
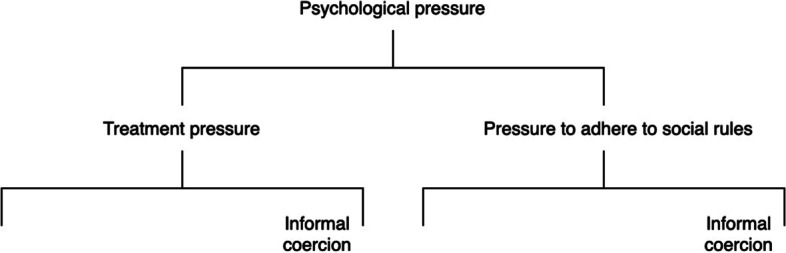
Conceptual relationships between psychological pressure, treatment pressure and informal coercion

These communicative strategies are discussed in the literature under the header “informal coercion” [1, 2]. A note on terminology is thus in order. Szmukler and Appelbaum [7] prefer the term “treatment pressure” over “informal coercion” for conceptual reasons. They argue convincingly that persuasion, interpersonal leverage and inducements need not involve coercion and that only pressures at the upper end of the spectrum do (see also [8, 9]). Accordingly, they propose to use the more neutral term “treatment pressures” to refer to the full spectrum. Anticipating our findings, we found that the communicative strategies outlined by Szmukler and Appelbaum are used not only to improve treatment adherence but also to improve adherence to social rules. As with treatment pressures, pressures to adhere to social norms need not involve coercion. For these reasons, we will henceforth use “psychological pressure” as an overarching concept encompassing both treatment pressure and pressure to adhere to social norms. Figure 1 depicts the conceptual relationships between psychological pressure, treatment pressure and informal coercion.

The use of psychological pressure is *prima facie* morally problematic because it can compromise the voluntariness of service users’ consent [10] or involves treating service users unfairly [11]. Szmukler and Appelbaum [7] rank the various communicative strategies hierarchically based on the level of pressure involved, resulting in a spectrum that ranges from persuasion at the bottom end to threats at the top end. The authors note that the use of pressure can be either morally permissible or impermissible, depending on the level of pressure and the strength of the justification one has for exerting it: the higher the level pressure, the stronger one’s moral justification must be.

Ample empirical evidence is available on formal coercion, particularly on its prevalence [12–14], stakeholders’ attitudes toward it [15] and the methods by which it can be reduced [16–18]. By contrast, the scientific evidence on psychological pressure is scarce and normative guidance for professionals is mostly lacking [1]. This is partly due to the lack of a clear definition and workable operationalization. Further exploratory and conceptual research is thus needed.

Various empirical studies have indicated that psychological pressure is common in mental healthcare services [1, 2]. Professionals in a large international focus group study identified both positive and negative effects of psychological pressure. Positive effects included enhanced treatment adherence, avoidance of decompensation and reduction of formal coercion; negative effects included an impairment of the therapeutic relationship and coercive stigma of services [19].

Survey and focus group studies with mental health professionals suggested that professionals often use psychological pressure unknowingly [19–22]. Moreover, the focus group study carried out by Valenti and colleagues [19] revealed a dissonance between professionals’ attitude and practice, with professionals disapproving of forms of psychological pressure that they continue to use in their daily routine. This points to the possibility of social-desirability bias in qualitative and survey studies on the topic with professionals. The lack of awareness and underreporting of psychological pressure in research studies at least tentatively suggests an unreflective use of psychological pressure in clinical practice.

Explorative quantitative survey studies indicated that professionals see the use of psychological pressure as a less restrictive alternative to formal coercion [20–22]. These studies also showed that professionals tend to underestimate the level of psychological pressure. The more that professionals approved of coercion, the more they tended to underestimate the level of pressure; and the higher the level of pressure, the higher the degree of underestimation [20–22]. If the burden of justification increases as one moves upwards in the hierarchy of psychological pressures, these findings tentatively suggest that professionals regularly use psychological pressures in unjustifiable ways.

Psychological pressures can be analyzed not only in terms of what is said, but also in terms of by whom, why, how, when and where things are said. The scope of Szmukler and Appelbaum’s conceptual model [7] is restricted to what is said by professionals to improve treatment adherence and does not consider the larger context of interaction. Sjöström’s [23, 24] notion of “coercion contexts” provides a promising theoretical framework for a more comprehensive analysis of psychological pressure. “The term context,” Sjöström notes, “serves to stress that coercion will carry different meanings for different actors in different situations” ([23] p41).

Several empirical studies that include service users’ perspectives indicate that the scope of analysis should be broadened to capture the full phenomenon. The inductive analysis of focus groups with mental health professionals carried out by Pelto-Piri and colleagues [25] suggested that the communicative strategies outlined by Szmukler and Appelbaum [7] should be supplemented with the strategies cheating, disciplinary style, and reference to rules and routines. Studies on perceived coercion underlined that service users’ perception of coercion depends not so much on isolated actions [26, 27] as on contextual and procedural factors, such as interpersonal relationships and procedural fairness [28–33]. Procedural fairness denotes the extent to which service users feel that they are being taken seriously and treated with respect [26, 34, 35]. In other qualitative studies on psychological pressure, service users reported pressure to adhere to recommended treatment and remain healthy from relatives and friends [25, 36], as well as from the dominant biomedical model of health and illness [23, 28, 35, 37].

The aim of our study was to develop a conceptual model of psychological pressure based on the experiences and perspectives of service users. Our research questions were as follows: (1) Which forms of psychological pressure do service users experience during inpatient stays and in their social environment? (2) How do service users evaluate these forms of psychological pressure? We expected that the analysis of the data would provide indications for potential strategies to reduce psychological pressure in mental healthcare.

## Methods

We chose an exploratory qualitative research approach and particularly the grounded theory methodology according to Strauss and Corbin [38, 39]. This enabled us to remain open to experiences of psychological pressure which have not yet been described in the literature and to develop a conceptual model of psychological pressure based on the perspectives of service users. Accordingly, we did not use the available models of psychological pressure as a basis for the interview guide. Instead, we asked participants open narrative questions about (a) their experiences of psychological pressure in their contact with mental health professionals or informal caregivers before, during and after stays in a mental health hospital; (b) their evaluation of situations in which they experienced psychological pressure; and (c) their suggestions for the reduction of psychological pressure in mental healthcare. Table 1 includes our guiding questions.

**Table 1:** Guiding questions

• You have experienced inpatient stays before. How did these stays come about and how did they proceed?
• Did you experience any situations that you associate with compulsion or psychological pressure? Could you tell us about these situations?
• If you think of the situations you described in which you experienced compulsion or psychological pressure, how do you feel about these situations?
• What do you think could have prevented your experience of compulsion or psychological pressure in these situations?

 The study received ethical approval from the Research Ethics Committee of the Medical Faculty of the Ruhr University Bochum, registration number 18-6584-BR.

### Sampling and data collection

The analysis draws on semi-structured qualitative interviews with 14 mental healthcare service users. Inclusion criteria were a self-reported psychiatric diagnosis and previous experience with formal coercion. Participants were not under involuntary commitment at the time of the interview. All participants were provided with detailed information about the study, both orally and in writing, and gave written informed consent prior to study participation.

We generated the sample by employing the theoretical sampling method, looking for a variety of dimensions arising from the first data analysis. These dimensions included age, diagnosis, number of involuntary hospital admissions, location of involuntary hospital admission and attitude toward the mental healthcare system. We contacted participants not only via mental healthcare services but also via independent self-help groups to ensure a variety of attitudes toward the mental healthcare system in the sample. The participants contacted via inpatient services were approached by one of the researchers who works as a psychiatrist at a mental health hospital. The researcher did not serve as the treating physician and made it explicit that participation could be declined without reprisal of any sort. The participants contacted via independent self-help groups approached us via email on their own initiative after we distributed information about the study among self-help groups across Germany.

The final sample consisted of 14 service users. The characteristics of the sample are described in Table 2. We describe the characteristics on a group level rather than an individual level to ensure the anonymity of study participants.

**Table 2:** Sample characteristics

Dimension	Description
age	21–63
gender	7 female, 7 male
self-reported diagnosis	6 psychotic disorders, 6 affective disorders, 1 substance dependence, 1 personality disorder
experience with coercion	ranging from one involuntary hospital admission to recurring involuntary admissions over the course of decades (including extensive experience with mechanical restraint and involuntary medication)
contact method	7 via inpatient services, 7 via self-help groups

All interviews were conducted in Germany between May 2019 and January 2020. The interviews lasted between 45 and 120 min and were carried out either at the participants’ homes or at a mental health hospital, depending on the preferences of participants. The interviews were conducted by SP, either alone or together with JG, CH or MS, and by JG together with CH. Twelve interviews were conducted by two researchers and two interviews by one researcher. Outpatient participants received 15 euros as reimbursement of time and other expenses.

All interviews were audio-recorded and then transcribed verbatim. The data analysis was carried out based on transcripts in the German language. The interview excerpts that are cited in the article were translated from German into English with the support of an English native speaker who is fluent in German. In translating, we aimed to preserve the meaning of the complete utterance rather than the literal translation of individual words. This sometimes required us to look for idiom and set phrases in the English language with a meaning similar to the original German idiom. All cited English excerpts were compared with the original German data to ensure that meaning was preserved. Punctuation was added on some occasions to improve readability.

### Data analysis

The grounded theory methodology according to Strauss and Corbin [38, 39] enabled us to adopt an open and inductive approach and simultaneously to incorporate our contextual clinical and theoretical knowledge in the data analysis. During the data collection and after the first interviews were conducted, SP and CH started open coding the data inductively, each researcher working on different transcripts. In an iterative process using axial coding, SP and CH adjusted and restructured the emerging codes by comparing codes within and across transcripts. Each researcher focused on transcripts open coded by the other researcher to ensure intersubjective traceability of the data. As a further step in the iterative process, SP and CH theorized the data by using selective coding and focusing on the data from the perspective of working assumptions resulting from the first analysis. In a final step, SP, CH and MS developed the conceptual model of psychological pressure by going back and forth between the working assumptions and the data. We used the software MAXQDA 2020 Standard (VERBI Software GmbH, Berlin, Germany) for the data analysis.

All steps in the iterative data analysis were discussed continuously between SP and CH, and both provisional and final results of the data analysis were discussed with AG, JG and MS to ensure intersubjective traceability and include interdisciplinary perspectives. Conflicts in the analysis were discussed either between SP and CH or with the full research team until consensus was reached. The research team included one researcher with a background in sociology, one in philosophy and three in medicine and medical ethics. All five researchers have clinical experience: one as a psychiatrist, one as a resident in psychiatry, two as auxiliary nurses and one as an intern.

## Results

**Fig. 2:**
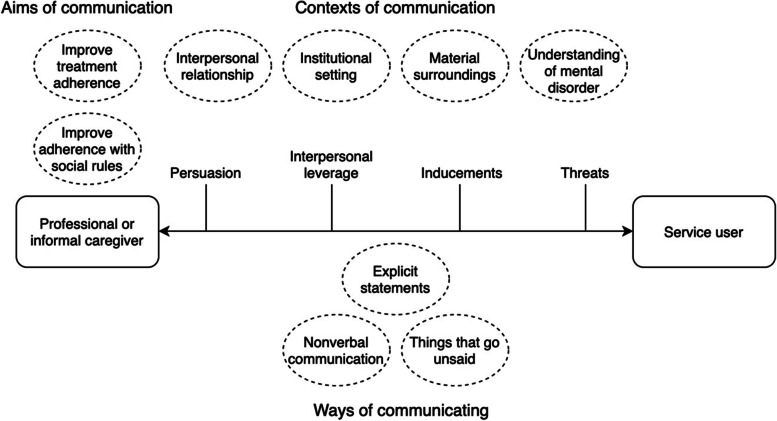
The contextual model of psychological pressure

We derived three main categories inductively from the data: the aims, the ways and the contexts of communication. These categories are presented below. The contextual model of psychological pressure is based on these categories. The analysis of the data showed that the aims, ways and contexts of communication influence service users’ perception of psychological pressure strongly. Two general aims of communication emerged from the data: communication aimed at increasing service users’ adherence to treatment and communication aimed at increasing their adherence to social norms. The data showed three important dimensions in the ways of communicating, namely, explicit statements, nonverbal communication and things that go unsaid. The data also revealed that the extent to which service users experience psychological pressure depends greatly on the context of the communication. Four relevant contexts of communication emerged from the data: the quality of the interpersonal relationship, the institutional setting, the spatial surroundings of communication and the level of convergence between service users’ and professionals’ understanding of mental disorder. The contextual model of psychological pressure which emerged from the analysis of the data is depicted in Fig. 2.

### Aims of communication

It emerged from our data that service users see professionals and informal caregivers as pursuing various aims when service users experience psychological pressure. These aims can be grouped into two general aims: improving service users’ adherence to recommended treatment and improving their adherence to social norms.

### Pressure to improve adherence to recommended treatment

The analysis of our data indicated that psychological pressure is used commonly to promote treatment adherence. Service users reported experiences not only with professionals but also with relatives and friends.

It emerged from our data that service users often experience information disclosure in the context of informed consent as involving more than an exchange of information about the expected benefits and risks of available treatment options. It often involves argumentative attempts to influence service users’ decision-making and motivate them to accept recommended treatment. This marks the difference between an exchange of information and persuasion. The following quote illustrates that persuasion can easily turn into authoritative communication:

*The first thing was that I was not allowed to leave the ward. I was surprised and I said, “What’s this all about? I can take a walk outside.” … “No you can’t, you can’t.” At that point, I became suspicious. Then I got these little pots for these drops or whatever they are called … tablets. […] Then I asked what they were and was told that it was none of my business. “You have to take them, and then you feel better.” And then I said, “I won’t take anything when I don’t know what it does to me.” Yeah, [they said] I shouldn’t ask such stupid questions.* (Service user 8)

Service users reported various experiences that involved interpersonal leverage. These experiences were reported primarily in interactions with relatives and friends. A striking example is the following statement:

*My* *mother said,* *if you are home alone, I cannot go to work because I am afraid that you will harm yourself … if you don’t go to the hospital, I’m worried.* (Service user 3)

Service users reported another set of experiences that involved inducements. These experiences were reported predominantly in interactions with professionals. Service users recalled professionals making statements like the following:

*If you participate in occupational therapy, you will receive a packet of tobacco.* (Service user 5)

Another service user told us that she would be discharged from the mental health hospital on the premise that she visits the outpatient services regularly:

*If I’ll go there regularly, I can live in the community and they will leave me in peace.* (Service user 14)

Another set of experiences involved threats. These experiences were reported across various contexts and in relation to both professionals and informal caregivers. One service user told of a professional on the ward who made the following statement:

*And if you’re not nice, then you’ll come here on the bed later and then I’ll strap you on with the things that I just attached to this thing here.* (Service user 13)

Service users also reported these experiences in the context of communication with relatives and friends in the community. One service user provided a vivid illustration of this:

*If I hadn’t gone to the hospital, my mother would probably have arranged an involuntary hospital admission.* (Service user 3)

### Pressure to improve adherence to social norms

Our data indicated that psychological pressure is used to increase adherence to not only treatment but also social norms. The latter type of psychological pressure was reported predominantly in relation to house rules concerning mealtimes, television times and wake up times. Other rules in relation to which service users typically experienced psychological pressure concerned smoking or drinking coffee or tea between meals. Service users particularly perceived communication about social rules as involving psychological pressure when the communication reinforced the power imbalance between staff and service users or the rules were perceived as arbitrary, unjustified or unnecessarily rigid. Service users described a strong influence of such rules on the atmosphere on the ward and in the mental health hospital, as becomes clear from the following quote:

*Well, it’s always like that, they always insist a lot on having this structure. […] I mean, getting up at half past six and especially when in the evening I’ve usually, well, I take Seroquel [quetiapine] once a day and that is in the evening. And if you take six hundred milligrams, then you’re rested after eight or nine hours. But sometimes you just can’t get up and moving and it’s also generally just hard for me … and to get up at seven and yes, that was always difficult. So, at the beginning, some of them are annoying and then wake me up and come back in, and here again and there again and there again.* (Service user 13)

The following quote illustrates how service users can experience communication about social rules when rules are applied rigidly and no justification is given:

*I was hungry once, late in the evening, which means 9 o’clock in the evening, and was really hungry and they also know that the pills make you hungry, they must know, and yes, I said, “I would like to have something to eat.” […] “There’s nothing left.” … I say, “What? I’m hungry.” … “There is nothing left.” I say, “Yes, so what should I do now?” … “Yes, uh, you’ll just have to endure.”* (Service user 12).

Another service user told of the use of inducements to improve adherence to social rules regarding wake-up times and cleanliness.

*Participant: This one ward X*_1_ *is completely closed, you have to earn a leave with points.*

*Interviewer: And how do you get these points?*

*Participant: Yes, by getting up on time, getting things done, ah, cleaning.* (Service user 2)

### Ways of communicating

When describing experiences of psychological pressure, service users tended to emphasize the importance of the way in which things are said. Dimensions in the ways of communicating were explicit statements, nonverbal communication and things that go unsaid.

### Explicit statements

Service users reported a wide variety of explicit statements in response to which they experienced psychological pressure. Forms of persuasion that were experienced as exerting a high level of psychological pressure included expressions of prohibitions, such as *“No, you can’t [do that]”* (service user 8); expressions of obligations, such as *“You have to take [these tablets]*” (service user 8); and imperatives, such as *“Stay here and sit down”* (service user 1). Explicit statements involving interpersonal leverage, inducements or threats typically took the form of hypothetical or conditional statements with an if-then structure. Examples are *“If you don’t go to the hospital, I’m worried”* (service user 3); *“If you participate in occupational therapy, you will receive a packet of tobacco”* (service user 5); and “*If you’re not nice, then […] I’ll strap you on”* (service user 13). Threats were also expressed as imperatives followed by a description of an alternative scenario in which the service user would be worse off: *“Don’t do that, or we’ll discharge you”* (Service user 10).

### Nonverbal communication

Under nonverbal communication we understand the transfer of information through facial expressions, bodily gestures, body posture and the like. The analysis of the data showed that nonverbal communication plays a role in whether service users perceive a communicative strategy as exerting psychological pressure. Psychological pressure was experienced primarily when professionals’ nonverbal communication suggested that service users were not taken seriously. One service user made a vivid contrast when relating about positive encounters with staff:

*That they … speak the same language when they talk to you, that they are not so condescending, that they can listen, that they tackle problems, don’t look arrogant.* (Service user 5)

Well-attuned nonverbal communication was perceived as a motivation to continue with treatment and as supportive in the recovery process. One participant told of a nurse who was particularly competent in the nonverbal aspects of communication:

*Participant: She was one of the people … where I noticed in my long medical history that she … she still conveyed that the illness is now perhaps acutely bad and stressful, but there is … recovery, and there are possibilities, and we help you with it.*

*Interviewer: How did this become clear?*

*Participant: So, it’s just to give you an idea. She never wore the very simple care clothes but had smocks with frills and so on. Always a little bit extravagant […], then the hair is pinned up, then with curls, then the eyes are always made up, very big eyes and accordingly made up. One had the feeling that big children’s eyes look at you. You know, how children have these round eyes and such. And the facial expressions, very active facial movements and nonverbal communication, and very patient-oriented.* (Service user 10)

### Things that go unsaid

The analysis of the data showed that psychological pressure can also arise when pieces of information are not communicated. This can even amount to deception when health professionals consciously leave out selective information during the information disclosure to lead service users into accepting recommended treatment. The following quote shows that deception and selective information disclosure are not only ethically objectionable, but probably also ineffective, because they can be counterproductive in enhancing treatment adherence:

*Another point is, the doctors don’t point out the side effects of the tablets at all, that you get so fat from them, yes, and you have to know that. I now have dress size 48. I had 38 before. That’s why I always stopped taking the tablets.* (Service user 12)

Threats involve conditional proposals to make service users worse off if they turn down the proposal. Professionals sometimes make proposals without making it clear what will happen when service users turn these down. It emerged from our data that service users sometimes perceived such proposals as threats. An explanation for this is that they inferred negative consequences attached to turning down the proposal from prior experience. This effect was amplified when service users experienced a strong dependency on the treatment team. One participant, for instance, described the following events and their effect on her:

*Participant: Then they came with, I don’t know, five, six, seven people or so. They didn’t touch her and then they stood around her, and then she voluntarily … she was not even aggressive and nothing like that … and then she voluntarily went into the staff’s room and let herself be restrained … and that really upset me back then, yes.*

*Interviewer: Now you have said that she went along voluntarily. If you think about it again, how would you describe voluntarily?*

*Participant: That voluntary? … Not really voluntary … voluntary in quotation marks, because I know about … other patients who even if they are in a psychosis, sometimes they would also approach them with six, seven people if they can’t get them to calm down. They get an injection and are then restrained, and this leaves traces not only on those who are subjected to it, but also on the other patients. If you observe something like this … or even just hear about it, it has an effect on you when you are a patient there, and I think the biggest problem is the helplessness you feel … that you cannot control any of this.* (Service user 9)

### Contexts of communication

Communication and interpretation always take place in a context. The analysis of the data indicated that the experience of psychological pressure depends on the context of interaction. Incorporating contextual elements into the model of psychological pressure allows one to understand why identical statements can be perceived as exerting pressure in one situation but not in another. Four contextual factors stood out as particularly influencing service users’ experience of psychological pressure: the quality of the interpersonal relationship, the spatial surroundings, the institutional setting and the level of convergence between service users’ and professionals’ understanding of mental disorder.

### The quality of the personal relationship

The data suggested that perceived psychological pressure depends on whether personal relationships with professionals or informal caregivers are experienced as supportive or discouraging. Discouraging interpersonal relationships were characterized by service users as involving a lack of transparency, a lack of emotional support, a feeling of being unknown to each other, unfair treatment and strong dependence. Communicative interactions in the context of discouraging relationships were more likely to be perceived as involving psychological pressure. One participant reported discouraging personal relationships with professionals in the context of an admission process:

*When I look back on it in retrospect, one could have said, “Mr. X*_1_, *there is something wrong with you, you are in a manic episode, you are out of line, you are … not in command of your powers, … or you are not sane at the moment … we have to keep you here and if you don’t want that, then unfortunately we have to restrain you.” Something like that, a clarifying conversation or something. That somehow didn’t happen at all. But it was all just like this, “Here, Mr. X*_1_, *now stay here and sit down and … let’s do something like this.” … Run-of-the-mill exchanges, according to the motto ‘The main thing is that we calm him down.’* (Service user 1).

Service users also described supportive personal relationships. The following factors seemed to have a positive effect on the quality of the relationship: time to get to know each other and build mutual trust, transparent advice at eye level, care and commitment despite illness-related behavior, not being reduced to one’s mental disorder and exchanges with peers or professionals with personal experience of mental health crises. Relating about her experiences on a ward where these conditions were fulfilled, one service user told us about an experience of psychological pressure in which the quality of the personal relationship played a key role. It turned what might have felt like coercion in different circumstances into a positive communicative exchange:

*Participant: They were all looking at me in a very friendly way and said to me, “Something’s not right, the way you are right now […] you are so agitated […] somehow we have the feeling that something’s not quite right. I think you’d better go home and see a doctor.” Then I said, “Yes, I’ll do so.” … When three, four people look at you in a friendly way and worry about you …*.

*Interviewer: Well, that sounds like it was something positive for you.*

* Participant: Yes, this caring attitude, yes, and not that kind of pressure like “No, don’t do that, or we’ll discharge you.” But it was pressure, […] I don’t know what would have happened if I had said, “No […] I will stay here, I don’t see it that way, I don’t want to.” That I don’t know. […] I think it has to do with the relationship I have with people, whether I have the feeling that they are well-disposed towards me.* (Service user 10)

Service users reported comparable experiences within the context of personal relationships with partners, relatives and friends, as the following quote illustrates:

*Participant: Well, it was somehow at night, and I couldn’t sleep, and he [my partner] said, “We’ll go to the clinic now,” and then we drove.*

* Interviewer: And so you agreed, didn’t you?*

*Participant: Uhm, he tried it the night before [laughs]. So, I called a buddy and asked my buddy to make it clear to him that it is not necessary yet, and he succeeded. And the next day, I called a friend with whom I talked about this earlier and whom I also involved. And I think she said, uhm, maybe it wouldn’t be a bad idea, or something like that. So, I think the two agreed with each other, and then I said, ok, then we’ll go.* (Service user 13)

### The institutional setting

The institutional setting is another factor that influenced perceived psychological pressure positively or negatively, depending on whether the institutional setting was perceived as supportive or hindering. A supportive institutional framework was described by service users as a protective and tranquil space that influences the interaction with professionals positively. Supportive interventions that were mentioned included open wards, single rooms and permission to leave a closed ward accompanied by staff or in a protected outdoor area. Other features that were mentioned were good care resources (in terms of staff, therapy options and equipment), relief from everyday obligations, and protection against relapse in relation to addiction, mental health crises, homelessness and loneliness. The following quote illustrates how a supportive institutional setting can reduce the perceived psychological pressure:

*Participant: I simply went there of my own accord, when my doctor said, “Maybe it would be good if you could take a rest for two weeks.” Then I did it voluntarily, coming here.*

*Interviewer: Oh, with rest he meant …*.

*Participant: Yes, switch off, shut down. I went to the doctor on my own initiative and thought I would go to the hospital here for another three to four weeks. People don’t hurt me. They have good intentions.* (Service user 5)

Some service users described the institutional setting as discouraging. Lack of options on the ward and unsuccessful cooperation between wards were mentioned in particular. One participant described how transfers to another ward can disrupt the continuity of care and influence communication with professionals negatively:

*There were always discussions about discharge. I just find it, well, I often had an order for six weeks. Sometimes it was brought to an end before … sometimes, sometimes not. And … then in place X*_5_, *I said okay, then I’ll just stay on this closed ward for these six weeks and after that I leave […] It is always at the point where you are transferred, then you get other doctors and other staff, they want to get to know you again. It takes another three weeks and that’s always something to consider, where I think, well, do I want to be moved again and then start all over again, or do I hold out for two more weeks and then go home directly?* (Service user 13)

Service users tended to describe closed wards in particular as institutional settings that hinder communication with staff, as the following quote highlights:

*In psychiatry, there are conditions that need a lot of improvement and especially in closed psychiatry. So when you’re in there, it’s really terrible that the door is closed and you’re not allowed out. I wasn’t allowed out for six to seven weeks and I walked up and down like a tiger in a cage, and I found it terrible and I find it terrible every time. They do have nurses there, but nobody really talks to you.* (Service user 12)

### The material surroundings

The analysis of the data indicated that the material surroundings of communication also influence perceived psychological pressure. The following quote shows exemplarily how the material surroundings of communication shape the meaning of communication, especially by influencing service users’ inferences about the consequences of noncooperation:

*Then I came into a room … was supposed to sit at the table there. First I got something to eat. It was around noon and that probably wasn’t a normal patient room, but it was, I don’t know if you call it an admission room, where there is actually no office, but a room, but with windows so that you can look inside. Where there was a table with chairs, but also a patient bench, and these belts were attached to this patient bench and they are used if necessary … but that was in there by default, and that irritated me because I thought, why is this patient bench there now or why is it there with these devices?* (Service user 10)

Material surroundings can have an influence on service users’ perception of the general atmosphere on the ward and attitudes of the staff:

*This respectful attitude is what is missing in hospital X*_2_ *for the most part, or actually is simply just missing. […] And then at some point I found myself there, and then … then I was on the closed ward for the first time, and there I said to the doctor “Why are there locks on the windows everywhere?” And then … I don’t remember what she said.* (Service user 9)

The service user continued to speak of other negative experiences she had had on the ward. When asked what it did to her, the impact on her perception of communication with the staff became clear:

*One becomes defensive. […] adapting … Let me put it this way, I just tell the doctors what they want to hear, I just say it casually now, because one is afraid. Every day something can happen, you know?* (Service user 9)

### Convergence between the parties’ understanding of mental disorder

The final contextual factor that influenced perceived psychological pressure is the level of convergence between service users’ and professionals’ or informal caregivers’ understanding of mental disorder. It was important whether service users presupposed a biomedical or a social model of mental disorder or denied the existence of mental disorder altogether. Clashes between models of service users and professionals tended to influence the meaning of communication strongly. If service users did not understand themselves as having a mental disorder, for example, biomedically-oriented professionals were more likely to be seen as exerting psychological pressure in conversations about diagnosis and treatment. Similarly, negative attitudes toward medication on the part of service users made it more likely that statements made by professionals were perceived as exerting psychological pressure. The following quote illustrates the convergence between models of mental disorder:

*I was also diagnosed for the first time during this time, because I was asking questions very insistently. … So I got my medication and, of course, I also thought about it for myself. Now I seem to have something that is not over with a single episode. Then I simply asked the head physician during the doctor’s round. I said, “Now, please just tell me, what is it that I have now?” And then the doctor X*_1_ *said, well, basically a very banal metabolic disorder in the brain as the basic diagnosis and endogenous psychosis from the schizophrenia spectrum was the official diagnosis back then. And I’ll say a little flippantly, that was a working hypothesis that I could make use of. … I started with information material, which was officially distributed by the hospital.* (Service user 10)

Service users who denied that they have a mental disorder, or who emphasized that they only experience mental problems for a limited amount of time, tended to see advice and treatment offers from professionals as involving psychological pressure. A possible explanation for this is that the claims made by professionals were seen as lacking adequate justification and are thus interpreted as involving psychological pressure rather than rational argumentation. Service users who understood themselves in this way also felt more often misunderstood by professionals. Such a clash of models of mental disorder is apparent in the following quote:

*We are not mentally ill, we are just different. Do you understand that there is a rejection of this term of illness, but that is problematic, because otherwise you have no access to the hospital there. You have to have a diagnosis and the cat bites its own tail again. Do you understand what I mean?* (Service user 6)

Another example of a lack of convergence is when professionals or informal caregivers interpret normal responses as manifestations of psychiatric symptoms, as the following quote illustrates:

*Then it was a situation like the one before with my mother, let me tell you, this hypersensitive attitude of hers. I find it a bit … because you have had it since you were 16, the illness, and thus have a history of psychosis. Then every little runaway becomes … you can’t do anything for your close relatives or anything else, you can’t even show a different image of yourself or try to change yourself. […] It’s just very small things and then it’s immediately, no, it is psychotic, psychotic, psychotic […] There is some coercion to it, I think.* (Service user 7)

The way service users come to grips with their disorder included various modes of self-regulation, such as control over one’s emotions, adherence to hospital regulations, flexibility in relation to treatment options, and competence in dealing with conflicts. The more service users felt they succeeded in these forms of self-regulation, the less likely they were to report the experience of psychological pressure during inpatient stays.

## Discussion

Our findings suggest that the most widely accepted conceptual model of psychological pressure, the one developed by Szmukler and Appelbaum [7], must be broadened and radically contextualized. Nevertheless, the data subsumed under the category “pressure to improve adherence to recommended treatment” confirmed Szmukler and Appelbaum’s model. This is an interesting finding considering the open and inductive design of our study. Though being familiar with Smukler and Appelbaum's model, we did not rely on it during the development of the interview or category guide for methodological reasons. When similar phenomena emerged as themes from the analysis, we decided to adopt Szmukler and Appelbaum’s terminology to avoid potential misunderstanding and conceptual confusion due to the inflation of terminology.

In line with Sjöström [23], our study highlights the relevance of “coercion contexts.” Our findings indicate that psychological pressure (1) is used to improve not only treatment adherence but also adherence to social rules; (2) is exerted by not only professionals but also relatives and friends; (3) is due to not only the content of communication but also the way of communicating; and (4) depends greatly on additional contextual factors, including the quality of the interpersonal relationship, the institutional setting, the spatial surroundings and the type of discourse. The resulting conceptual model defines psychological pressure comprehensively in terms of what is said by whom, why, how, when and where. This context-sensitive model of psychological pressure can explain why service users can experience comparable communicative interactions negatively and as involving psychological pressure in one situation, and positively and as supporting in another.

Other studies also stress the contextual nature of treatment pressures [23, 25, 28, 34–36, 40]. Pelto-Piri and colleagues [25] concluded that the communicative strategies of persuasion, interpersonal leverage, inducements and threats must be supplemented with new categories, such as disciplinary style and referring to house rules and routines. Larsen and Terkelsen [40] and Norvoll and Pedersen [34] also found that psychological pressure is exerted to improve not only treatment adherence but also adherence to social rules. During the inductive analysis of our data, we judged that it was not necessary to create additional categories at this level. Although we found phenomena closely related to those described by Pelto-Piri and colleagues, the difference was not so much in what was said as in why it was said. References to house rules were typically made by means of the communicative strategies already described by Szmukler and Appelbaum [7], only with a different aim, namely, to improve adherence to social rules rather than recommended treatment.

Our study confirmed the findings reported by Canvin et al. [36] and Pelto-Piri et al. [25] that not only professionals but also relatives and friends exert psychological pressure on service users. The relevance of the way of communicating to the perception of psychological pressure was also found in other studies. Pelto-Piri et al. [25], for example, highlighted the relevance of disciplinary style and Verbeke and colleagues [35 brought to the fore the relevance of silence and lack of communication. These phenomena are closely related to the phenomena that we subsumed under nonverbal communication and things that go unsaid. Further contextual factors that emerged from our data were found in other studies as well. The study carried out by Norvoll and Pedersen [35] underlined the importance of the personal relationship and the study by Larsen and Terkelsen [40] emphasized the importance of the material surroundings. Other studies yielded findings closely related to our finding that the level of convergence between concepts of mental disorder influences service users’ perception of psychological pressure [24, 28, 35, 37].

### Strengths and limitations

An important strength of our study is that it develops an empirically informed conceptual model of psychological pressures in mental healthcare based on the perspectives of service users. A limitation of our study is that we did not carry out a triangulation of the data: the analysis is based on the narrative data from interviews with service users and does not draw on other data sources, such as observations or interviews with professionals. The broader study included interviews with informal caregivers, but the results of this part of the study will be published separately.

We have consciously refrained from ethically evaluating the various factors influencing the perception of psychological pressure, as our aim was to understand what psychological pressure is and how it comes about. Nevertheless, by generating hypotheses about the origins of psychological pressure based on the perspectives of service users, our study takes a first step toward the development of ethical guidelines and training programs on the use and prevention of psychological pressure in mental healthcare.

### Implications for practice

Based on our findings, it is possible to draw the following implications for the use and reduction of psychological pressure in mental healthcare. Because the use of threats is ethically objectionable, professionals are advised to refrain from making conditional proposals to make service users worse off should they turn down the proposal. Considering that professionals may issue threats unknowingly, the examples of such conditional proposals reported in our study can enable professionals to reflect critically on their ways of communicating. The findings of our study also invite professionals to reflect on their ways of communicating more broadly, including their nonverbal communication (e.g. facial expressions, eye contact, gestures and bodily posture) and the type of discourse they use (e.g. biomedical or recovery-oriented discourse). They also highlight the importance of transparent communication. Considering that service users may infer negative consequences attached to non-cooperation from previous experiences, it will often be advisable to make explicit that service users can decline treatment offers without reprisal.

The findings of our study speak in favor of making deliberate choices about the place of communication, especially during the admission process and conversations about topics where resistance on the part of service users is likely. Similarly, the treatment team should consider the quality of the therapeutic relationships of individual staff members with service users and make deliberate choices about which staff member communicates with a service user in a difficult situation. Taken together, these implications underwrite the importance of developing training programs to enhance staff communicative competency with the aim of reducing the use of psychological pressure.

Furthermore, the results of our study suggest that changes at the organizational level may be effective in reducing psychological pressure. Considering that many service users report psychological pressure regarding social rules, a fairly simple way to prevent psychological pressure is to reduce the number of house rules to the minimum necessary for living together. Our study also brought to the fore that structural organizational problems influence perceived psychological pressure. Addressing these problems is thus likely to promote service users’ sense of voluntariness. Given that professionals experience discomfort in relation to the use of psychological pressure, addressing these problems is also likely to promote staff satisfaction. A final way to reduce the use of psychological pressure on the ward would be to cultivate an openness among staff members toward a diversity of concepts of mental disorder, care concepts and personal coping strategies.

## Data Availability

We cannot share the research data publicly as this may compromise the privacy of research participants. The anonymised data can be made available by the corresponding author upon reasonable request.
